# The Applicability of a Drop Penetration Method to Measure Contact Angles on TiO_2_ and ZnO Nanoparticles

**DOI:** 10.3390/nano10061099

**Published:** 2020-06-02

**Authors:** Sabrina M. Garner, Edgar A. O’Rear, Sharukh Soli Khajotia, Fernando Luis Esteban Florez

**Affiliations:** 1 Stephenson School of Biomedical Engineering, University of Oklahoma, 173 Felgar St., Norman, OK 73019, USA; smgarner@ou.edu; 2 Chemical, Biological, and Materials Engineering, IBEST, University of Oklahoma, 100 East Boyd Street, T-301, Norman, OK 73019, USA; 3 Department of Restorative Sciences, The University of Oklahoma Health Sciences Center College of Dentistry, Division of Dental Biomaterials 1201 N. Stonewall Ave., Oklahoma, OK 73117, USA; Sharukh-Khajotia@ouhsc.edu (S.S.K.); fernando-esteban-florez@ouhsc.edu (F.L.E.F.)

**Keywords:** nanoparticle, contact angle measurement, drop penetration method, powder, zinc oxide, titanium dioxide

## Abstract

In this study, six solvents (water, diiodomethane, bromonaphthalene, formamide, ethanol and ethylene glycol) were examined for three nanoparticle substrates, zinc oxide and titanium dioxide (21 nm and 100 nm), with the goal of assessing the suitability of a modified drop penetration method (DPM) for orders of magnitude smaller particles. Nanoparticles were compressed into flat discs and the solvent dropped on the surface while the image with time was recorded. Contact angles were in reasonable agreement with literature over the range of 20–80°, but failed to provide acceptable results for surface energy components. It was necessary to eliminate certain solvents and substrates not meeting the selection criteria.

## 1. Introduction

Nanoparticles (NP), which are discrete structures displaying high surface area-to-volume ratios, have a wide variety of applications in several segments of industry, engineering and health-care including paints, coatings, catalysts, cosmetics and pharmaceuticals [1,2,3,4,5,6,7,8]. In the medical field, NP are typically used for the fabrication of antibacterial coatings, nanostructured composite materials and implants [9,10] with metal oxides of silver, zinc and titanium [9,11,12,13] being the most commonly used due to their proven antibacterial and biocompatibility properties. Because metallic oxides are known for their antibacterial properties, their incorporation into a dental adhesive is of particular interest for this research on surface properties. The surface character of the nanoparticles is important to ensure good wetting and integration of the nanoparticles into the adhesive without compromising bonding function. In general, NP are mechanically dispersed (e.g., orbital planetary mixer, spatulation and ultrasonication) within the matrix of different types of materials (e.g., metals, ceramics and polymers), and their functionalization levels may be adversely impacted by agglomeration phenomena and strong interfacial effects. Therefore, understanding the surface properties of nanostructured powders may facilitate the development of novel nano-filled materials displaying improved properties (e.g., chemical, mechanical and biological) and may even help to predict their performance during service.

Properties such as roughness, hardness, bond polarity and electronegativity typically dictate how materials interact with organic and inorganic molecules, water, human tissues (both hard and soft) and cells (eukaryotic and prokaryotic), thereby directly influencing the biological and fouling properties of nano filled materials. Several studies have shown that the interaction between different types of solvents (such as water, alcohol, acetone) and solid surfaces, can be used along with contact angle measurements and the work of van Oss–Chaudhury–Good (vOCG) [14,15,16] to determine the surface free energies of a different surface.

**Figure 1:**
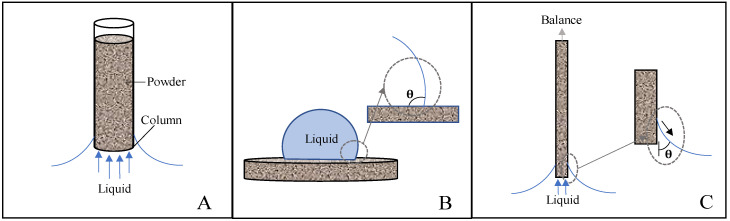
Common methods for wettability determination using contact angles. (**A**) Washburn capillary rise, where a capillary tube is packed with a solid and the liquid taken up is related to the wettability of the powder. (**B**) Static contact angle, where a liquid droplet is placed on a solid and the contact angle is determined from the liquid’s point of contact. (**C**) Wilhelmy plate, where a plate is inserted into a liquid and removed, the surface tension force is balanced by the force needed to pull or push the solid into or out of the liquid.

Measurement of powders’ contact angles is known to have its challenges. To date, there have been a number of methods developed to determine the wettability of powders [17], with much of the work being reported by the pharmaceutical industry. The Washburn capillary rise (WCR) method illustrated in Figure 1A uses a glass tube or column packed with powder to correlate the rate of liquid uptake (in terms of mass) to contact angle measurements [17,18]. There also exists a variation on WCR known as thin layer wicking with the powder deposited on a substrate [19]. Due to its simplicity and low cost, WCR stands out as the most commonly used method to determine the contact angles of powders. Despite such popularity and wide spread use, the WCR method has been demonstrated to be restricted in regards to the types of systems that can be measured, therefore, researchers have investigated the possibility to adapt the static contact angle and Wilhelmy plate techniques [17] (Figure 1) for accurate determination of powders’ contact angles in WCR-restricted systems. 

When reviewing the various methods for contact angle determination on compact powders, there are some key advantages and disadvantages to consider for each (such as cost, time, reliability and accuracy). For example, WCR is not appropriate for determining the wettability of hydrophobic particles using water as capillary forces will not allow the fluid to advance [20]. In addition, WCR method is also not suitable for particles that swell or packings that collapse during testing. According to Ramirez-Flores the packing procedure is a critical determinant of reproducibility [21] with channeling and wall effects being well-known problems that further impact the ability of WCR to be used effectively. Notwithstanding these concerns, the WCR method represents a convenient method for determining the contact angles of powders. Moreover, it should be appreciated that particulate substrates complicate the use of the static contact angle and Wilhelmy plate techniques which are generally employed for finding the wettability of monolithic materials with flat surfaces. 

The static contact angle (SCA) technique depicted in Figure 1B is typically carried out in a goniometer by dispensing a liquid droplet onto a solid surface, followed by the analysis of the drop profiles and the determination of wettability (in terms of contact angles) using the Laplace–Young equation. In comparison to other available methodologies, the static contact angle is one of the most efficient when cost and time are considered; however, its major disadvantage is associated with its limited accuracy in determining contact angles that are smaller than 20° [17]. These problems are further exacerbated by surface roughness, whether it is a loose layer of powder or a pressed disc. It has been demonstrated that variations in these surface properties can cause the Laplace–Young contact angle to more closely resemble the apparent contact angle, thereby adversely impacting the ability to measure the wettability of a powder [7,21].

The Wilhelmy plate (WP) method is an interfacial tension analysis that is calculated based on the observation of advancing and receding contact angles that are observed as the substrate (nonporous, thin, rectangular and flat) investigated is immersed into a liquid and then pulled back out into the position where the substrate (plate) makes first contact with the liquid. During this process, the advancing and receding contact angle measurements are obtained by taking the surface tension into consideration and observing the force. Even though WP is more expensive because of the requirement for the utilization of a precision microbalance, this method has the advantage of being capable to precisely measure small contact angles [22]. Based on these properties, researchers have adapted the WP method for powder-based specimens deposited onto the surfaces of wafer or glass slides and fixed using an adhesive [17]. Potential sources of error are related to the uncertainties associated to values of the wetted perimeter as directly influenced by surface roughness [17] and the possibility of added weight through the absorption of liquid into the porous substrate.

Recently, Liu et al. introduced a modified approach to the Drop Penetration Method (DPM) and applied it to determine the wettability of powders displaying particle size-distributions between 10–100 μm [23]. This method has been proven to work effectively on measure the contact angles on micron sized particles; however, it would be beneficial if this method could also be used on nanoparticles. The Washburn method has been used for determining the contact angle of nanoparticles; however, this method, as noted above, is associated with issues such as channeling. The DPM is a promising technique that will not have wall effects that contribute to channeling issues. DPM is a technique where digital images are captured to provide a time-dependent assessment of the penetration of the liquids (reference solvent and investigated solvent) into wafer specimens fabricated from compressed powders. This approach combines elements of the SCA and WCR methods, wherein a droplet is dispensed onto a solid surface of interest and advancing contact angles can be observed while the liquid is drawn into the porous material by capillary action. Despite these similarities, DPM differs from the SCA and WCR methods when it comes to data processing and experimental output (in terms of contact angles’ numerical values). Moreover, the SCA yields apparent contact angles while the DPM reveals the actual contact angles [17]. A significant difference between the WCR and DPM methods is that the former investigates liquid penetration opposed by gravitational forces while the latter works with gravity. 

Based on this critical scenario, the purpose of the present study was to examine the applicability of the DPM method reported by Liu et al. for the measurement of contact angles of powders composed of nanoparticles with dimensions that are 2–3 orders of magnitude smaller than those investigated by Liu et al. A wide range of solvents was employed in the present study to allow the determination of their contact angles on powders of nanoparticles of zinc oxide and titanium dioxide. Contact angle results obtained in the present study (modified-DPM) were compared to contact angle values previously reported in the literature for particles of similar compositions and size-distributions as determined using a variety of methodologies.

### Theory 

**Figure 2:**
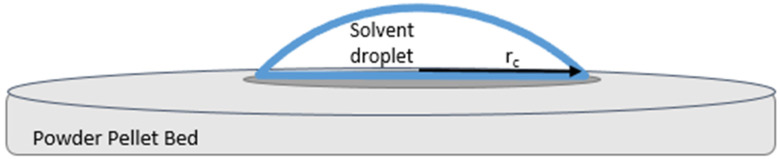
View of a sessile drop as the liquid penetrates into the dry nanoparticle bed and begins wetting the surface using the drop penetration method.

The derivation of the Drop Penetration Method and its equations involved are explained in detail by Liu et al. [23]. Scaling and nondimensionalization of the governing equations led to a relationship for the contact angle, as presented below by the final derived equation:

(1) $$\cos\left(\theta_{T}\right)=\frac{\tau_{\alpha R}}{\tau_{\alpha T}}\frac{\mu_{T}}{\mu_{R}}{\left(\frac{r_{\mathrm{cT}}}{r_{\mathrm{cR}}}\right)}^{2}\frac{\gamma_{R}}{\gamma_{T}}$$

where *τ*_α_ represents the penetration times, *μ* represents viscosity, *r*_c_ represents the contact radius, *γ* represents surface tension, and subscripts R and T represent the reference liquid and test liquid, respectively [23]. Subscript α indicates the particular fraction of the droplet volume that has penetrated into the disk for the value of τ. The reference liquid must have a zero-contact angle with the powder under consideration, that is, complete wetting cos(*θ*_R_) = 1. In addition, the contact radius of the drop must remain constant throughout most of the penetration for both test and reference solvent (Figure 2). 

## 2. Materials and Methods

### 2.1. Nanoparticle Powders

The metal oxide nanoparticles investigated in the present study were zinc oxide and titanium dioxide. Two types of commercially available zinc oxide nanoparticles have been tested: Nanogard^TM^ (Lot: B13Y045, Alfa Aesar, Ward Hill, MAilHHil) and NanoTek^TM^ (Lot: D22W010, Alfa Aesar, Ward Hill, MA, USA). Nanogard^TM^ nanoparticles are 40–100 nm in size and >99% purity. The NanoTek^TM^ nanoparticles are similar at 40–100 nm in size and 99% purity, but they differ in being hydrophobic with an organosilane coating. Commercially available titanium dioxide nanoparticles of two sizes were examined as the second type of metal oxide nanoparticles. The first of the titanium dioxide nanopowders contained 21 nm diameter particles comprised of a rutile and anatase mixture (Lot: MKCD9677, Aldrich, St. Louis, MO, USA). The second titanium dioxide nanopowder had larger particles (<100 nm), also containing a mixture of rutile and anatase (Lot: MKCG0376, Aldrich, St. Louis, MO, USA). The nanoparticles were used ‘as received’.

### 2.2. Fabrication of Specimens

A stainless-steel mold (25 mm I.D.) was used to fabricate flat disc shaped specimens composed of compacted nanoparticles’ powders (either ZnO [with or without organosilane coating] or TiO_2_). A pilot study was conducted to determine the necessary masses (1.4 g of ZnO and 1.0 g of TiO_2_, respectively) of nanoparticles required to fabricate specimens with thicknesses that did not allow the complete penetration of a solvent droplet through to the bottom of the disc during CA measurements. Because the DPM is based on interaction of the solvent with particles, if the solvent penetrates completely through the disc to the other side, another interface is introduced to the testing environment. This is similar to wall effects in the Washburn method. To determine the necessary thickness, one considers the volume of the droplet, porosity, and horizontal spread of the solvent in the disc. Nanoparticles were placed into the cavity of the mold, were evenly distributed to produce wafers of similar porosity and roughness, and were subjected to 140 MPa (2 min, 25 °C) of compressive forces delivered by a manual press (Specac Atlas 15T hydraulic press). The rationale for the selection of compressive forces described was based on previous published reports [17,24,25] indicating that the utilization of compressive forces between 70 to 800 MPa resulted in specimens displaying acceptable compression and mechanical properties without adversely impacting the nanoparticles (e.g., textures, morphologies and structures). Fabricated specimens were then carefully removed from the mold and stored in Petri dishes (dry and dark conditions) until use. 

### 2.3. Solvents

Six solvents (distilled water [lab grade], diiodomethane [Lot: S7359453, MilliporeSigma, Billerica, MA, USA], bromonaphthalene [Lot: S6971210, MilliporeSigma, Billerica, MA, USA], formamide [Lot: 94011020, Roche, Indianapolis, IN, USA], ethanol [Lot: SHBK0402, Sigma-Aldrich, Sheboygan Falls, WI, USA] and ethylene glycol [Lot: SHBK3427, Sigma-Aldrich, St. Louis, MO, USA, were selected based on their potential ability to meet the requirements of a reference liquid (e.g., complete wetting) and were used to determine the CA of specimens’ surfaces using the modified DPM method. Table 1 describes the viscosity, surface tension, and density properties of solvents investigated.

### 2.4. Contact Angle Measurements

**Figure 3:**
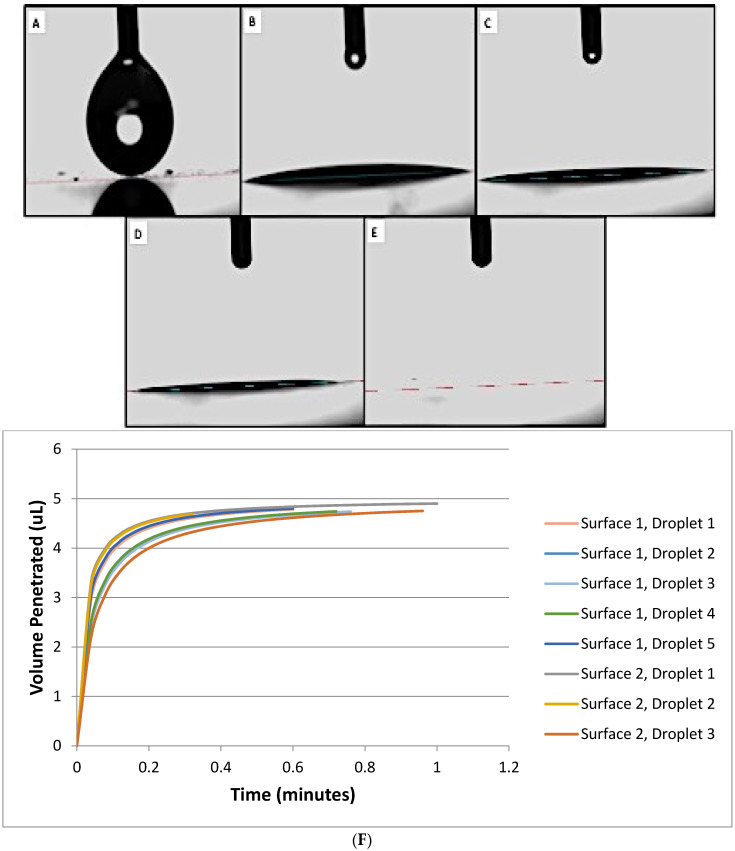
(**A**–**F**): Representative images of formamide on Nanogard^TM^; (**A**) formamide droplet immediately before being dispensed onto the surface of the specimen, (**B**) spreading of formamide droplet, (**C**) aspect of droplet after 0.50 min, (**D**) aspect of droplet after 0.75 min, (**E**) aspect of the surface of the specimen at 1.00 min Note that the droplet dispensed is completely absorbed by the specimen over the observation period of time. (**F**) Representative curves of time-dependent penetration of formamide (volume dispensed here) into specimens of packed Nanogard powders (either x or y). Surface numbers indicate separate specimens, while droplet numbers indicate separate droplets placed on the surface of same specimen.

A contact angle goniometer (OCA 15, Future Digital Scientific Corp., Westbury, NY, USA) coupled with a high-definition and high-speed digital camera (up to 50 frames/s) was used to measure the contact angle of a droplet. Samples were placed in a temperature controlled experimental chamber for measurement. A computer-controlled solvent-dispensing system was used to dispense individual axisymmetric droplets of each solvent (volumes according to Table S1) onto the surfaces of separate specimens (n = 2/group) at 3 random locations. Digital images (25 frames/s) were captured (1 min, 22 ± 1 °C) to determine the evolution of the drop absorption by the compressed disc. The evolution of the droplet and its contact angle was determined using OCA 15EC, SCA 20 V.4.4.3 software program. The average of the contact angle measurements at *τ*_α_ = 0.50 and *τ*_α_ = 0.75 are used for contact angle data reporting. It is important to highlight that even though drop-volumes varied according to solvent considered, volumes used never exceeded the threshold of 10 μL to prevent gravitational forces from distorting the results [28]. The volumes of droplets were adjusted due to the considerable variability in physical properties of the solvents. This allowed the droplets to dispense consistently. Figure 3A–F illustrates representative images of the evolution of a droplet penetrating into a specimen fabricated using nanopowders. Each droplet dispensed was observed to exhibit a circular cross-section in shape during penetration. The volume of droplets dispensed were calculated using Equation (2), below, for a spherical cap.

(2) $$\mathrm{Volume}=\frac{1}{3}\times \pi \times h\;\left(3\times a^{2}-h^{2}\right)$$

where h represents the height of the spherical cap and a represents the radius.

## 3. Results and Discussion

The modified-DPM was employed in the present study to determine the wettability of deionized water, bromonaphthalene, formamide, diiodomethane, ethanol and ethylene glycol (in terms of contact angles) on the surfaces of specimens fabricated with packed nanopowders of Nanogard™ ZnO or TiO_2_ to assess the utility of the modified-DPM for nanostructured materials. In order for the modified protocol to render accurate contact angle measurements, some fundamental criteria must be met, including: (i) the reference solvent’s contact angles must tend to zero and exhibit complete wetting; (ii) the contact radius should remain constant throughout penetration time; (iii) nondimensional time and volume (for different solvents on the same solid) should create an overlapping trend when graphed (Figure 3). The nondimensional time and volume take the permeability, porosity and effective pore radius into account in order to determine the nondimensional values of the data shown in Equation (3).

(3) $$t_{c}=\frac{\mu \varepsilon r_{c}{}^{2}}{kp_{c}}$$

where *t*_c_ is the characteristic time, *μ* is viscosity, *ε* is the porosity, *r*_c_ is the contact radius, *k* is the permeability, and *p*_c_ is the capillary pressure inside of the powder bed. More details on the nondimensional analysis equations can be found in the article by Liu [23].

In the present study, the rationale for selection of nanoparticles was based on their utilization as antibacterial and bioactive agents for polymer-based dental biomaterials, while solvents were selected based on their ability to determine the surface free energy components of the solids of interest [16], and because this type of characterization requires the utilization of a variety of solvents displaying a variety of affinity behaviors. Experimental results indicated that the solvents investigated were incapable of wetting the surfaces of specimens fabricated using NanoTek^TM^ and, therefore, failed to meet the first criterion cited. This resulted in exclusion of NanoTek from the present study. Table S2 lists the solvent used as the reference solvent for each contact angle analyzed. In addition, solvents investigated were observed to display droplets’ radii that were nearly constant at *τ*_α_ = 0.50 and *τ*_α_ = 0.75, thereby fulfilling the second criterion cited. The data measured at these times are used to calculate the contact angle results. The volume and height of the droplet do change over time as each solvent penetrates specimens investigated. Even though these characteristics are integral to determining the contact angles of the materials of interest, the nondimensional time and volume for different solvents on the same solid should create an overlapping trend when graphed. The results reported in present study were observed to fulfill this criterion completely (as seen in Figure 3), thereby suggesting that, despite intrinsic limitations associated to the utilization of solvent-specific drop volumes, satisfactory levels of accuracy and robustness were attained with the modified-DPM, which allowed us to perform intra-group comparisons.

**Figure 4:**
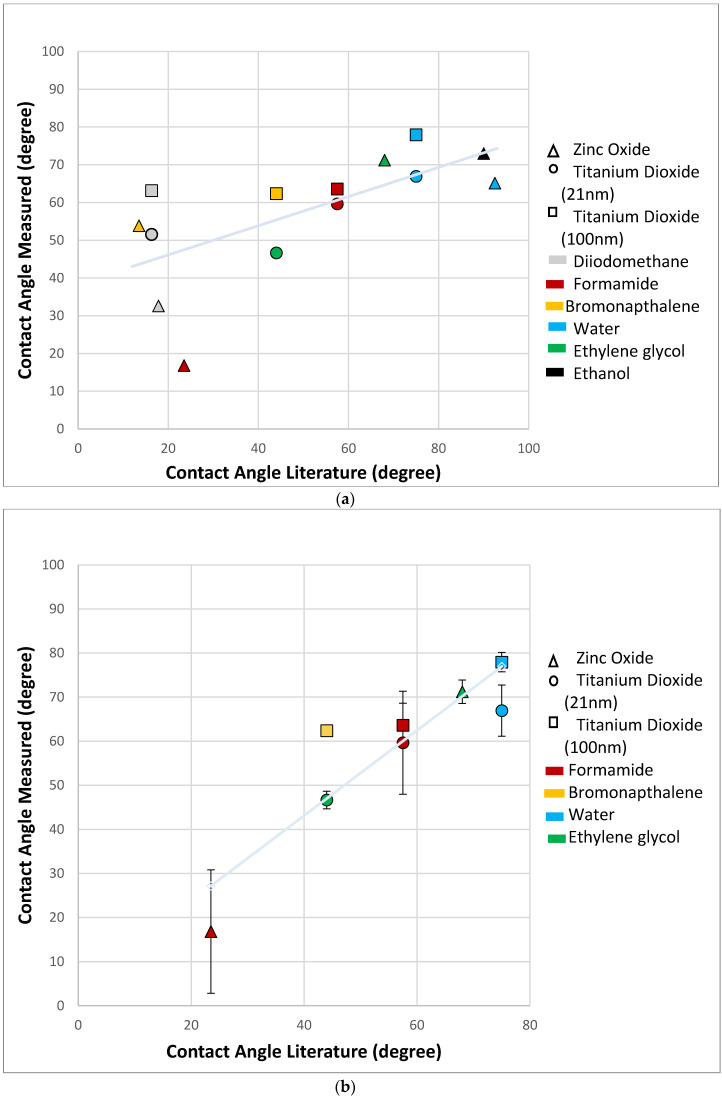
(**a**) Experimental contact angle versus literature contact angle based on substrate and solvent. A linear relationship can be seen in the data with the exception of points at low contact angles. The correlation value is 0.44 with a slope of 0.39 shown by the linear regression (light blue line). Symbol shape represents the nanoparticle tested and color indicates the solvent for the data point. (**b**) Experimental contact angle versus literature contact angle with elimination of data >90 degrees and diiodomethane. A clear linear relationship can be seen giving a correlation value of 0.82 and a slope of 0.97 shown by the linear regression (light blue line). The literature values are an average of the contact angle values using different measurement methods on a variety of surface types.

The experimental data obtained revealed that the modified-DPM proved to be most effective when measuring contact angles between 20° and 80° (Figure 4a). When the contact angles approached 90 degrees (poor wettability), the solvent experienced the weak capillary forces that are necessary to promote solvent penetration. This behavior not only impeded the recording of penetration time, but it also indicated that the modified-DPM cannot be used to assess contact angles accurately for some specific solvent-material combinations. Experimental data from solvents displaying this type of behavior have been excluded from the present study because $\tau_{\alpha T}$ is very large, $\cos\left(\theta_{T}\right)$ is zero and all such samples yielded contact angles of 90°.

The results obtained using the modified-DPM for solvent displaying contact angles smaller than 20° (e.g., diiodomethane) were not considered accurate because these solvents displayed significant coefficients of variation and were not comparable to values previously reported in the literature (Table S2). These findings are in agreement with a previous report by Alghunaim et al. [17] that while investigating several techniques for determining contact angle and wettability of powders, have indicated that the SCA method also display limited performance for accurately determining contact angles that are below 20°. Diiodomethane is a highly volatile solvent which is likely the reason it is not suitable for this testing method. It is possible that modified-DPM was able to discern differences with diiodomethane and the substrates, though this seems unlikely. Figure 4b shows the results from the correlation analysis (R^2^ = 0.82), between experimental (excluding results >90° and diiodomethane) and literature data. The line fit shown was obtained using least squares regression; it indicates the presence of a strong, positive and linear (slope = 0.97) relationship between these two datasets, thereby further corroborating the present study’s rationale for the selection of solvents and nanoparticles.

When examining which reference solvent would show promising results, it was found that the viscosity and surface tension of the test solvent and reference solvent were very important. If the value calculated using Equation (4) is ≤1, it is likely the reference and test solvent will be compatible and provide a reasonable contact angle data. All data with values ≤ 1 for this study returned reasonable contact angle results. If the value from Equation (4) was >1 the reference solvent and test solvent combination usually proved unsatisfactory for the contact angle measurement.

(4) $$\Pi =\frac{\mu_{T}}{\mu_{R}}\frac{\gamma_{R}}{\gamma_{T}}$$

Previous studies [29,30,31] investigating the effects of particle size and shapes on the wetting properties of metallic micropowders have indicated the presence of an inversely proportional relationship, wherein particles of smaller sizes were associated with higher contact angle values. The results of the present study are not in agreement with previous reports. A correspondence between TiO_2_ particles of smaller dimensions (diameter ≈ 21 nm) and lower contact angle values was observed; however, the data is not extensive and therefore cannot be conclusive. This finding could be partially explained by significant variations in capillary forces that result from materials fabricated using particles of different sizes and the effects of crystalline structure (e.g., rutile, anatase or brookite) as particle sizes decreases. One study [28] reported that rutile has a somewhat higher surface energy than anatase which suggests it should have a low contact angle with water [32,33]. Conversely, Pantaroto et al. while investigating the antibacterial efficacy of different crystalline phases of TiO_2_ photocatalysis against oral multispecies biofilms, found that rutile surfaces tended towards hydrophobicity (𝜃 = 95°) while anatase or a mixture of anatase and rutile showed an intermediate hydrophilicity (𝜃 = 90°), indicating a low surface energy [34]. That said, according to multiple studies, the properties of titanium dioxide will change under certain conditions of temperature or pressure and is dependent on particle size [5,35,36]. In this direction, even though anatase is more stable when particles sizes are small (typically below ~14.5 nm), increased pressures may cause anatase to become increasingly unstable [32].

The pressure used to compress the powders into discs has been found to affect the observed contact angles of compressed powders when assessed using common methods. Studies investigating the wettability of different types of materials using SCA or WPM have shown that contact angles tend to reduce as compression forces increase until a constant contact angle value is achieved [7,22]. Other studies [7,17] have shown that high compressive forces may plastically deform nanoparticles depending on their physical structure and their chemical compositions. The macroscopic manifestation of this behavior may adversely impact the penetration of solvents into materials by changing interparticle spacing, pore connectivity and changing capillary forces [7].

The greatest limitation to obtaining accurate measurements with the modified-DPM was directly related to the selection of solvents displaying contact angles smaller than 90°. Despite this significant limitation when the criteria for use were met, the method reported provided values that are comparable to those reported in the current literature [19,37,38,39,40,41]. As discussed above, there are different methods available to determine solvents’ contact angles on the surfaces of specimens fabricated using compressed powders. Table S3 exemplifies many of the commonly used methods for contact angle measurement and the typical values of contact angles obtained from the utilization of each method described. Once the contact angle values have been obtained, the van Oss–Good–Chaudhury technique can then be used to determine the surface free energy of the investigated surface; however, the vOCG technique has limitations when using the modified drop penetration method. The vOCG technique needs solvents with a variety of characteristics. When using the modified drop penetration method, the characteristics of the solvent are limited because they must completely penetrate the surface which means it should give a contact angle between 20° and 80°, therefore limiting solvent choices and making the use of the vOCG technique somewhat unfavorable.

**Figure 5:**
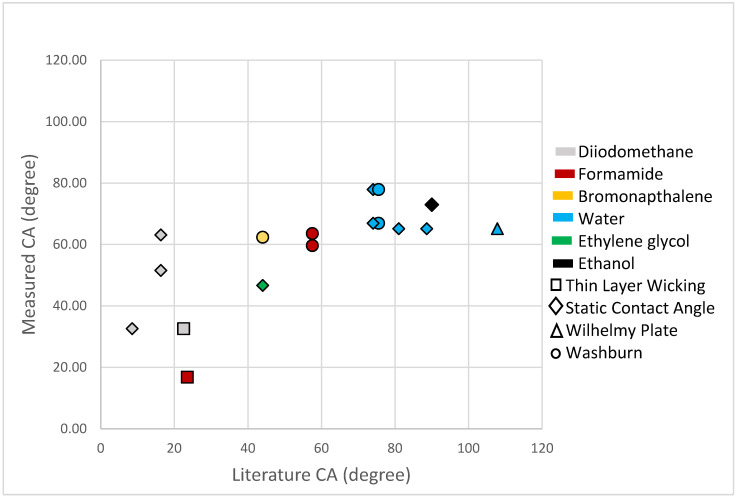
Comparison of the modified-DPM experimental values and literature contact angle values based on the type of technique used. The results have indicated that an obvious correlation between the experimental and literature contact angle values with any particular method could not be made. (The thin layer wicking method is a variation of the WCR method with powder deposited on a substrate.) Ideally, values would fall on a line with a slope equal to 1. This figure illustrates the challenge in obtaining reliable contact angles. Symbol shape represents measurement technique and color the solvent used for zinc oxide and titanium dioxide substrates.

When reviewing the experimental data reported in the present study and literature values, it can be seen that a clear correlation cannot be found between the modified-DPM and the accuracy of the experimental results obtained (Figure 5). The surfaces in which the literature contact angle values were measured on varied across the studies reviewed where insufficient contact angles data on compact powders was observed; therefore, literature values typically reflect contact angle measurements performed on solid specimens as well as on the surfaces of specimens fabricated using compacted powders.

## 4. Conclusions

In summary, the modified-DPM is a good technique for determining contact angles on lyophilic surfaces of nanoparticles when solvents are expected to display contact angle values varying between 20° and 80°. This method can be easily learned and uses inexpensive equipment that are typically found in surface chemistry laboratories; however, if accurate measurements using a variety of solvents are made necessary, a different technique may be necessary. This method is valuable due to the ability to measure the contact angle of nanoparticles with various solvents. This method can then be applied and used in the vOGC method to determine surface free energy of the nanoparticle granted the solvents meet criteria. The greatest impediment to use of the modified-DPM is identification of solvents that fit the criteria required for the adequate use of the modified-DPM. It is difficult to predict which solvents will fit the criteria before testing the solvent on the test surface. The most promising way to determine reference and test solvent compatibility is by using the parameter Π defined by Equation (4). If Π ≤ 1, it is very likely that the reference and test solvents are compatible and will give a quantitative contact angle measurement when using the modified-DPM.

## Figures and Tables

**Table 1:** Liquid properties of solvents at 25 °C.

Solvent	Viscosity × 10^3^ (Pa·s)	Surface Tension × 10^3^ (N/m)	Density × 10^3^ (kg/m^3^)
Diiodomethane	2.6	50.8	3.33
Formamide	3.34	58	1.13
Ethylene glycol	16.2	48	1.11
Bromonaphthalene	4.8 ^a^	44.4	1.48
Water	0.89	72.8	1
Ethanol	1.095	22.4	0.79

Note: All data cited from Van Oss et al. [26] unless denoted otherwise, ^a^ [27].

## Supplementary Materials

The following are available online at https://www.mdpi.com/2079-4991/10/6/1099/s1, Table S1: Summary of dosing volume and rate for solvents in CA goniometer, Table S2: Summary of contact angles determined from the modified-DPM, Table S3: Summary of methods used for contact angle measurements in literature.
