# Supplementary material for: The Applicability of a Drop Penetration Method to Measure Contact Angles on TiO2 and ZnO Nanoparticles
Source: Nanomaterials (Basel). 2020 Jun 2;10(6):1099. doi: 10.3390/nano10061099 (PMC7353180; doi:10.3390/nano10061099)
Supplement: Supplementary file 1 [file nanomaterials-10-01099-s001.pdf]

Supplementary Material

The Applicability of a Drop Penetration Method to Measure Contact Angles on TiO_2 and ZnO Nanoparticles

Sabrina M. Garner [1], Edgar A. O'Rear [2,*], Sharukh Soli Khajotia [3] and Fernando Luis Esteban Florez [3]

[1] Stephenson School of Biomedical Engineering, University of Oklahoma, 173 Felgar St., Norman, OK 73019, USA, smgarner@ou.edu
[2] Chemical, Biological, and Materials Engineering, IBEST, University of Oklahoma, 100 East Boyd Street, T-301 Norman, OK 73019, USA
[3] Department of Restorative Sciences, The University of Oklahoma Health Sciences Center College of Dentistry, Division of Dental Biomaterials 1201 N. Stonewall Ave. Oklahoma, OK 73117, USA; Sharukh-Khajotia@ouhsc.edu (S.S.K.); fernando-esteban-florez@ouhsc.edu (F.L.E.F.)
* Correspondence: eorear@ou.edu; Tel.: +1-405-325-4379

Received: 28 April 2020; Accepted: 28 May 2020; Published: date

Table S1. Summary of dosing volume and rate for solvents in CA goniometer.

Solvent	Dosing volume (μL)	Dosing rate (μL/s)
Deionized water	3	2
Bromonaphthalene	3.5	2
Formamide	5	3
Diiodomethane	1.5	2
Ethanol	4	2
Ethylene glycol	3	2

Table S2. Summary of contact angles determined from the modified drop penetration method.

Powder	Measured Solvent	Reference Solvent	Experimental Contact angle	Literature Contact angle
Zinc Oxide Nanogard	Diiodomethane	Bromonaphthalene	35 ± 8	17, 28 [19]
		Ethylene glycol	30 ± 3	
	Formamide	Bromonaphthalene	17 ± 14	25, 22 [19]
	Ethylene glycol	Bromonaphthalene	73 ± 1	60.4, 75.6 [37]
		Diiodomethane	69 ± 4	-
		Formamide	72 ± 3	-
	Water	Bromonaphthalene	79 ± 2	88.6 [38], 81 [39]
		Diiodomethane	77 ± 1	
		Ethylene glycol	47 ± 11	
		Formamide	78 ± 1	
		Ethanol	44 ± 7	
	Ethanol	Bromonaphthalene	75 ± 3	90 [40]
		Ethylene glycol	71 ± 1	
		Formamide	73 ± 1	
Titanium Dioxide (21nm)	Diiodomethane	Bromonaphthalene	75 ± 3	16.3 [41]
		Ethylene glycol	81 ± 2	
		Formamide	62 ± 1	
		Ethanol	34 ± 21	
	Formamide	Bromonaphthalene	56 ± 6	50–65 [18]
		Ethylene glycol	71 ± 4	
	Bromonaphthalene	Ethylene glycol	62 ± 1	38–50 [18]
	Water	Bromonaphthalene	84 ± 2	70–81 [18]
		Diiodomethane	67 ± 5	
		Ethylene glycol	86 ± 1	
		Formamide	79 ± 2	
		Ethanol	73 ± 1	
	Ethanol	Bromonaphthalene	68 ± 10	
		Ethylene glycol	77 ± 6	
		Formamide	48 ± 12	
Titanium Dioxide (100nm)	Diiodomethane	Bromonaphthalene	61 ± 5	16.3 [40]
		Ethylene glycol	43 ± 13	
	Formamide	Bromonaphthalene	71 ± 8	50–65 [18]
		Diiodomethane	49 ± 13	
		Ethylene glycol	60 ± 14	
	Ethylene glycol	Bromonaphthalene	47 ± 2	38–50 [18]
	Water	Bromonaphthalene	82 ± 1	70–81 [18]
		Diiodomethane	73 ± 1	
		Ethylene glycol	78 ± 2	
		Formamide	60 ± 10	

	Ethanol	41 ± 15	
Ethanol	Bromonaphthalene	78 ± 4	-
	Diiodomethane	66 ± 5	
	Ethylene glycol	72 ± 7	
	Formamide	49 ± 4	

Table S3. Summary of methods used for contact angle measurement in literature.

Surface	Liquid	Method	Contact angle
Zinc Oxide	Diiodomethane	Thin-layer wicking method	17, 28
		Static contact angle	8.5
	Formamide	Thin-layer wicking method	25, 22
	Ethanol	Static contact angle	90
	Water	Static contact angle	88.6
		Static contact angle	81
Titanium Dioxide	Diiodomethane	Static contact angle	16.3
	Formamide	Washburn Method—Rutile	50–65
	Bromonaphthalene	Washburn Method—Rutile	38–50
	Water	Washburn Method—Rutile	70–81
		Static contact angle	74
	Ethylene glycol	Static contact angle	44
